# Speculum Vaginæ

**Published:** 1850-11

**Authors:** 


*Speculum Vaginæ.*—Quite a warm discussion appears to be going on in Great Britain respecting the use of the Speculum in the treatment of vaginal and uterine diseases. We have selected for the Eclectic Department of this No. of our Journal two articles on the frequency of the affections requiring the use of this instrument, one by Dr. Tyler Smith, and the other, a rejoinder, by Dr. Bennet. Dr. Bennet, as our readers need not be informed, is the author of a work on uterine affections, which has led to the more frequent use of the Speculum than formerly with British and American Practitioners. We had supposed it was generally conceded that in this particular the practice of medicine had received an important improvement, and that many females are now promptly and effectually relieved, who, under the old system of treatment without the advantage of ocular investigation, were doomed to an indefinite continuance of distressing ailments. Certain are we that we have derived great assistance from the use of the Speculum since Dr. Bennet's book appeared in this country; and we have some cases to report, when our leisure will permit, which will serve to illustrate its great value in the management of uterine diseases. An impartial reader of the discussion reported in the last No. of Ranking's Abstract, (Report on Midwifery, etc.,) can hardly repress suspicion that the ardor of opposition on the part of Drs. Robert Lee and Ashwell, both authors of works on the Diseases of Females, springs more from personal feeling toward Dr. Bennet, than from that excessive regard for delicacy which they affect. The views of Drs. Acton, Locock, and the Editor of the Report just referred to, will, we think, commend themselves to the good sense of most medical readers.

